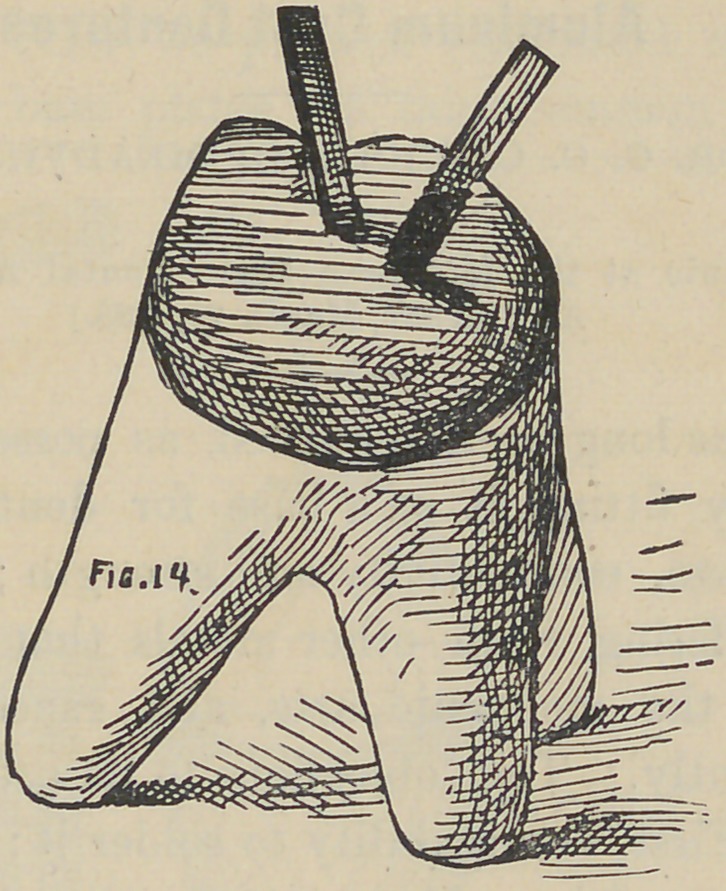# Porcelain Crowns

**Published:** 1888-06

**Authors:** E. C. Moore

**Affiliations:** Detroit, Mich.


### Porcelain Crowns.

BY DR. E. C. MOORE, DETROIT, MICH.

[Read before the Michigan State Dental Association, held at Ann Arbor, Mich., March 20, 1888.]

By reference to your programme, you will see that I am down for a paper, and to prevent disappointment I will announce to you beforehand that my paper is of a descriptive character, describing as briefly as possible a few illustrations, which I have endeavored to make so plain that not only he who runs may read, but even he who does not spend the time to read may understand from the illustrations; hence this apparent apology. If I fall short of the mark you may lay it to either procrastination or want of actual time in taking more deliberate aim at the mark, for this matter principally has been arranged since last Saturday evening. I don't usually delay these matters so long, therefore this hint is dropped so that those charitably inclined will interpret it as an apology for defects.

Crowns of various kinds have received deserved attention from the profession for several years past, and this combined effort on their part has resulted in great good in the way of repairing defects in human machinery. I say deserved attention, since we have demonstrated by actual test that otherwise useless or even more than useless roots have been made a thing of beauty and joy for a long time if not forever, roots which have been a source of irritation and pain to that extent that they are presented to us only because of this pain, the patient coming for their removal as a last resort for relief. Such teeth or roots I say, are often restored to usefulness, beauty and comfort by proper treatment, and the attachment of some non-irritating mechanical fixture which will articulate with the occluding tooth, thus bringing this otherwise useless root into use and exercise, and consequent health and comfort.

My illustrations and descriptions are for the purpose of showing how porcelain may be used in connection with crown work to make it more artistic, and demonstrate other advantages. First of which is the reduction of complicated operations to simplicity; second, setting a crown or half crown without being obliged to devitalize the pulp; and third, we are at liberty to set screws in the roots wherever the natural make of the tooth affords their strongest anchorage regardless of the relative position of one screw to the other, and it is a well known and established fact in mechanics that nothing holds stronger or better than a screw, and especially where more than one screw is inserted and at different angles. And by this process of crowning we can avail ourselves of this advantage, as my illustrations will show.

I will describe first the simpler form and perhaps the one we most frequently have to make, that of the bicuspid, and in this we are supposed to be dealing with a bicuspid root, decayed or broken considerably beneath the gum, with the nerve canal properly cared for. The next thing to do will be to remove all decay from the exposed end of the root down to hard tooth bone, no matter if the surface of the end of the root is left quite irregular by such removal, all the better in fact. The next move will be the setting of a screw or post as shown in Fig 1. Cut off to suit articulation, now slip a thin tubing of iridio-platinum over this screw of proper length, next cut a piece of very thin platinum of sufficient size to slightly more than cover the end of the root as prepared, with the rubber punch, punch out a hole in the center of this plate somewhat smaller than the tubing, slip this over the tubing, which is already in position over the screw, in so doing the edge will be slightly cupped up but nicely filling the tube; burnish this plate to fit the uneven end of the root and sufficiently over the edge or periphery to mark its boundary; dry the surface of the metal and put on a mixture of plaster and white sand and when sufficiently hard remove, and should the little plate not come away with the investing and tube, remove it afterward when it will readily go to place on the investing. Proceed at once to solder by placing a few small pieces of pure gold in the little depression on the plate about the tube; when soldered drop into a little water when the investment will crumble away; then with a pair of surgeon's shears trim away as indicated by the burnishing over the periphery of the root, when the piece will resemble Fig 2. Now slip this skeleton back to place again on the root; the soldering process having softened the metal, complete the burnishing until satisfied with adaption. The next step will be to take an impression. This is most satisfactorily done in plaster. If the vacancy be bounded by a tooth on either proximate side, then the impression must extend to these teeth. To govern the size of tooth to use, and if the space between these teeth be such that the impression will not draw—for it should be left to become quite hard—then this emergency must be met by making the impression sufficiently thick laterally, and trimming down on the grinding end so that it may be split in halves with a thick bladed knife, and the halves removed in opposite directions, and the skeleton removed from the root and placed in its proper position in the impression; and instead of fastening the two halves of the impression together with shellac or any kind of varnish, hold the two halves in position between the fingers and bridge over the break with a plate of softened wax, caught at each end with a little melted wax from the spatula. Next place a piece of German silver wire, of size to fit the tubing, down the tube, leaving the end projecting $\frac{1}{2}$ or $\frac{3}{4}$ of an inch and fill the impression; when hard remove the wax and the two halves of the impression are easily removed leaving the skeleton occupying the same position as in the mouth. The proper tooth having previously been selected, and for this the writer prefers the "How crown," the recommendation being its shape, length and position of pins, and as occasion requires remove either top or bottom pins, adjust to proper position on the model and flow hot wax to the tooth and skeleton to secure to place; when the wax is cold, split the model through where the German silver wire is inserted and invest in plaster and sand, leaving the German silver wire projecting beyond the investment, Fig 3, No. 1; when hard remove wax, bend pins around the tubing to suit; the wire having filled its mission, that of preventing the crushing of the tubing by bending the pins about it, is removed by carefully rotating with a pair of pliers. When the investment is properly dried over the Bunson burner, a little solder is placed about the pins and soldered fast. I say a little solder, because the less solder the better the porcelain will adhere to the metal; the rest of the process is as easily done as described; it consists simply in packing the porcelain about the pins and tubing where desired, placed in the gas furnace and baked. When the piece presents the appearance shown in Fig. 4, the darkened portion representing the added porcelain.

Fig. 5 represents a molar tooth, and with a good portion of undecayed tooth substance projecting beyond the gum, with the decay all removed and the edges prepared to get the greatest

Fig. 4.

Fig. 5.

strength. Fill in the center with cement, or plaster will do, (for either only serves a temporary purpose), until it presents the appearance of Fig. 6. The light line *k*, being the tooth substance and eventually to be the bearing for and joint of the porcelain and tooth, *b* is the cement or plaster. At this stage an accurate impression is taken in plaster, when hard remove and trim prop-

Fig. 6.

Fig. 7

erly, and without getting a model, get the imprint of this impression in Melotte's Moldine, make a die and counter die; strike up a little plate or cap to fit the tooth, as shown in Fig. 8. The two pins *b b*, are the open ends of a staple which passes through the cap, and a little bowl-like piece *a* in Fig. 7, placed on the opposite side of the plate from that shown in Fig. 8, which is easily made by cutting out with an ordinary belt punch on a piece of lead and soldered to the cap, together with the staple as shown in Fig. 7, *a b*, and is designed to hold the crown to place on the tooth by being imbedded in the cement, and *b b*, in Fig. 8, to hold the porcelain to the plate, the dotted line indicating the porcelain when complete. The temporary cement having answered its purpose is now removed and fresh added, and the crown permanently cemented to place. To make these three pieces *a b* and *m* one, invest the cap *m* in plaster and sand, punch two holes in the little bowl *a*, the proper distance to receive the staple *b*, place it on the cap *m*, pierce through into the investing with small drill,

Fig 8

Fig. 9.

and without removing the drill, do the same with the remaining hole, then remove both, drop the staple *b* to place, and solder together as one piece.

Fig. 9, simply shows the ordinary form of crown, where a small portion only of porcelain is required for grinding purposes. The telescope is made in the usual way, removed from the tooth with the plaster impression. A model made, remove the telescope from the model and cut out the plaster from the grinding end, say an $\frac{1}{8}$ or a $\frac{1}{16}$ of an inch from the edge of the telescope, fit in a thin piece of platinum to fit nicely, and two or more threaded platinum wires placed wherever desired, and the whole soldered to place. This being done, and the porcelain being baked to suit the taste, small square nuts may be put upon the threaded end of the wire, these to firmly hold in the cement when placed on the tooth; the screws in the tooth being treated in a similar manner, producing a very strong appliance, viewed mechanically.

Fig. 10

Fig. 11

These nuts are quickly made by cutting a strip from heavy German silver plate about an $\frac{1}{8}$ of an inch wide, punch with holes about the same distance apart with the plate punch, and tapped, with the shears snip off as required. If a round nut is preferred, make a tubing of suitable size, tap with the ordinary tap, slit the end with the small saw for the screw-driver or wrench, and saw off; these nuts are useful in various ways.

And now, gentleman, the next crown we are about to construct, differs from the first or bicuspid crown, chiefly as regards the number and position of the screws, one to the other, which you will observe, by following the natural direction of the canals in the roots, will of necessity throw the screws at different angles, as Fig. 12 shows. To proceed with the *modus operandi*, the roots are drilled but not tapped, at least not at this time, if at all, this however at the discretion of the operator. The writer would recommend never to use any other but the twist drill for drilling the root where a screw is to be placed for crowning purposes.

The next step is to fit the plate over or on the end of the root, by either burnishing or swaging, governed by circumstances. The plate being fitted and in place, two holes (or more), are made through it, corresponding to those drilled in the root; next slip wires through these holes in the plate and into those drilled in the root, to fit snugly, but not too tight; next the tubing is slipped over these projecting wires to connect with the plate; this of course places the tubing in line with the hole drilled in the root, now build the plaster and sand about these tubes and well over the surface of the plate and allow it to become quite hard, next draw the wires, carefully lift the whole from the tooth with the tubes and plate in their relative position; solder as before described, remove investing, solder brace joining tubes together, and in case of any change of positions of the parts, place back upon the tooth again and drop the wires back to position; this will align the parts if any disarrangement has taken place. At this stage the appearance is like that shown by Fig. 13. The wires are now withdrawn and the crown completed by building the body or porcelain to suit, and baked as shown in Fig. 14 complete; and in position on the tooth the wires left to show the relative positions of the tubing in the crown, and the position of the

Fig. 14.

screws (if screws are to be used) in the tooth or roots. If thought desirable the roots may be tapped, and screws in place of plain wire used, but before the crown is finally adjusted, a little wax may be placed at the orifice of the opening to the root, and the tubes in the porcelain crown served in like manner; this simply to prevent the cement from filling them. This can easily be removed through the tubes in the porcelain crown, and the screws driven home. I think you will all agree with me that the mechanical make up of such a crown is sound.

I have endeavored to make my description brief, and at the same time, plain as possible without going too much into detail, and in my eagerness to do so may not have made some important points plain; and if so, and you will be kind enough to ask any questions, I will cheerfully do what I can to enlighten you.

## Figures and Tables

**Fig. 1. f1:**
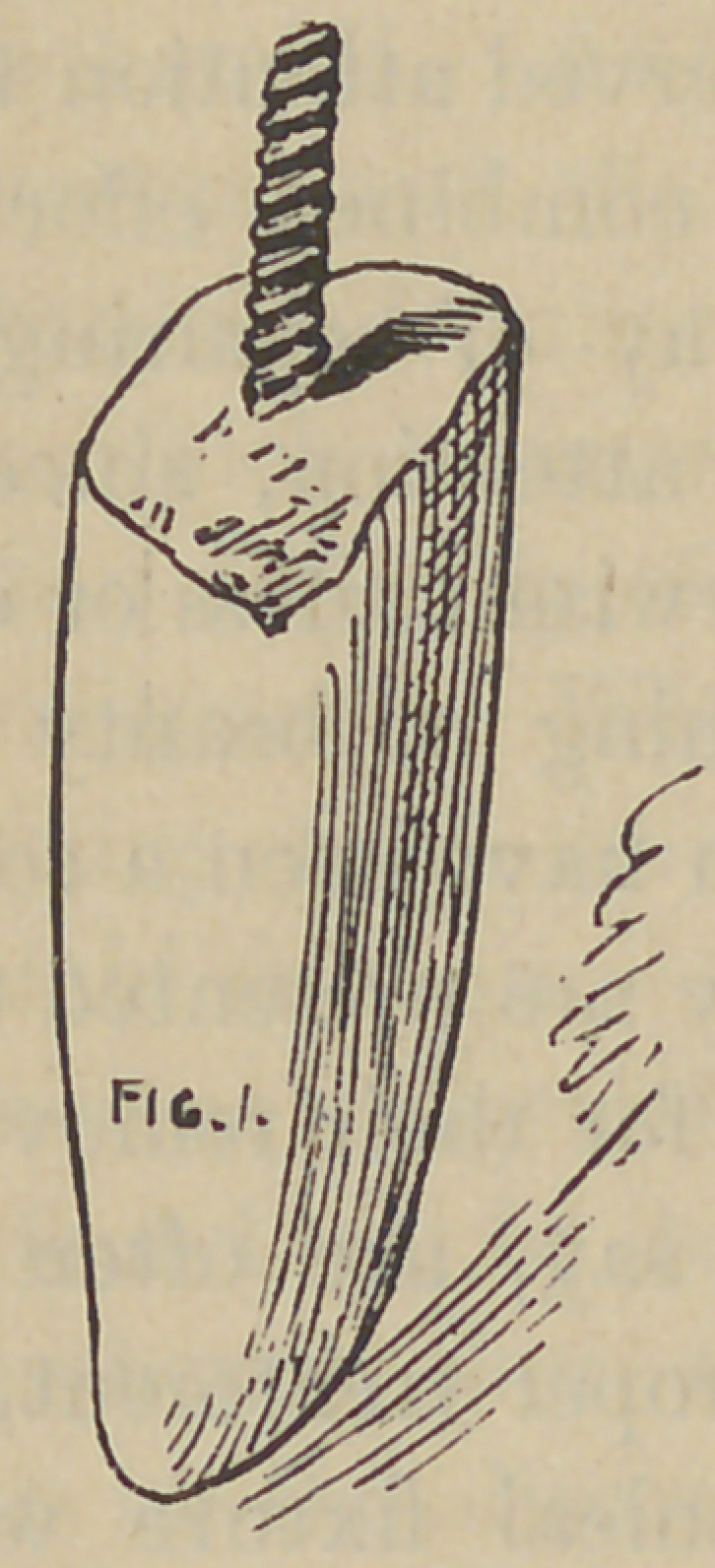


**Fig. 2. f2:**
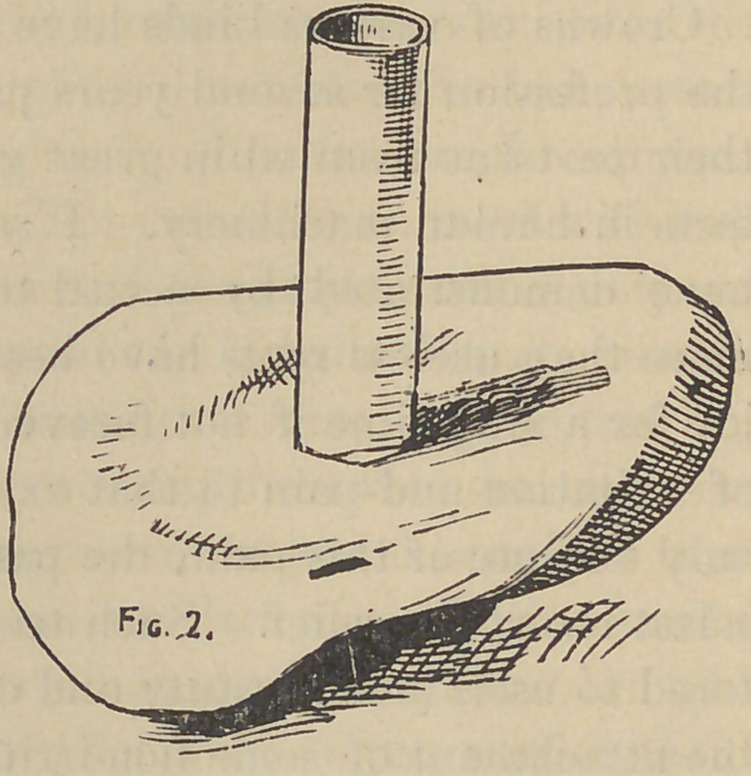


**Fig. 3. f3:**
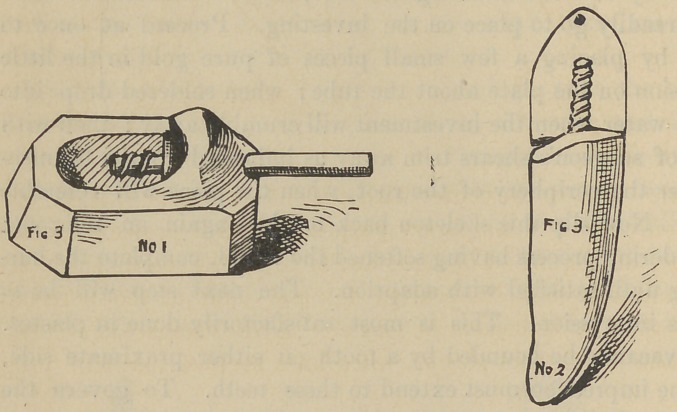


**Fig. 4. f4:**
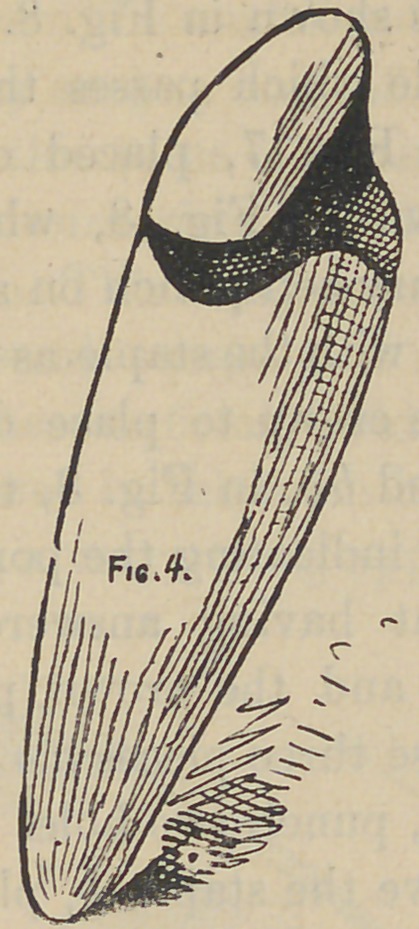


**Fig. 5. f5:**
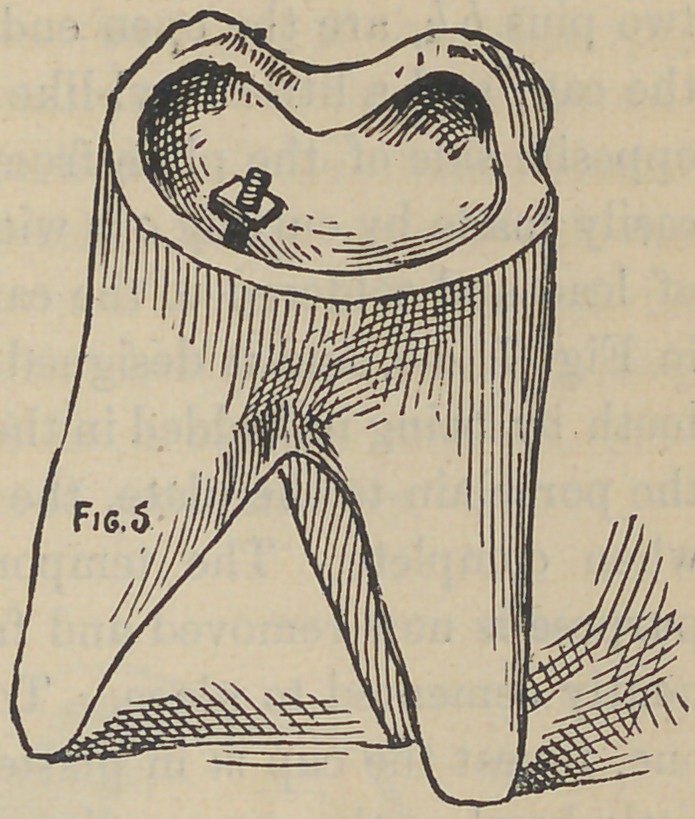


**Fig. 6. f6:**
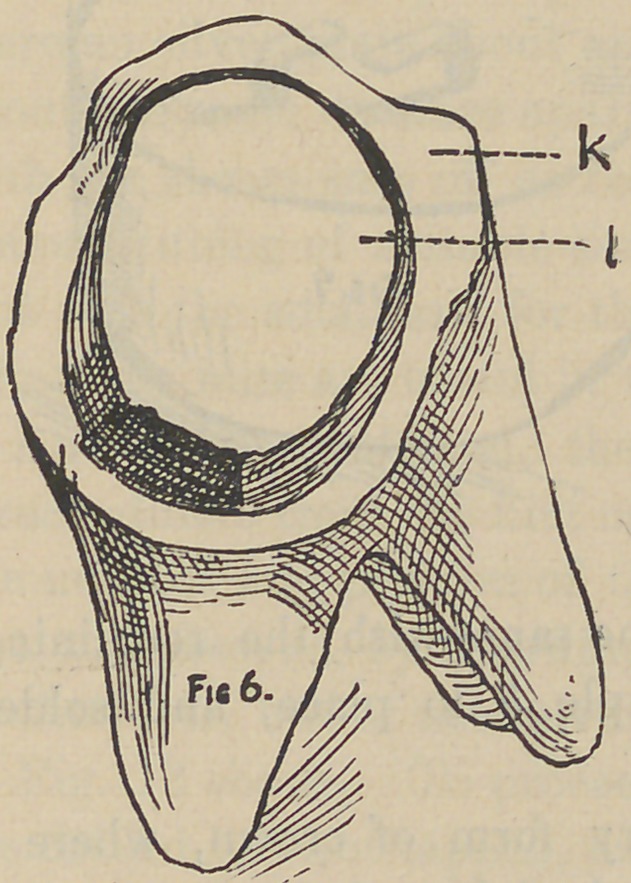


**Fig. 7. f7:**
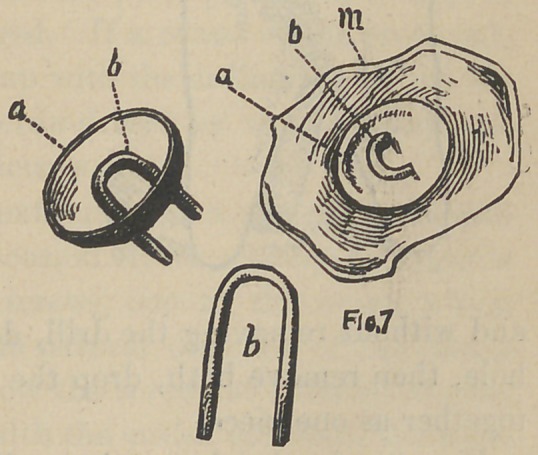


**Fig. 8. f8:**
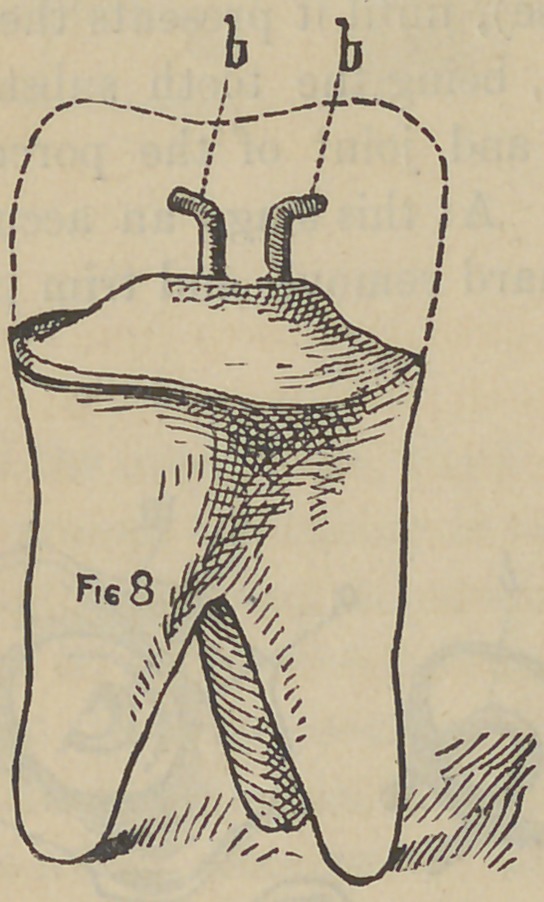


**Fig. 9. f9:**
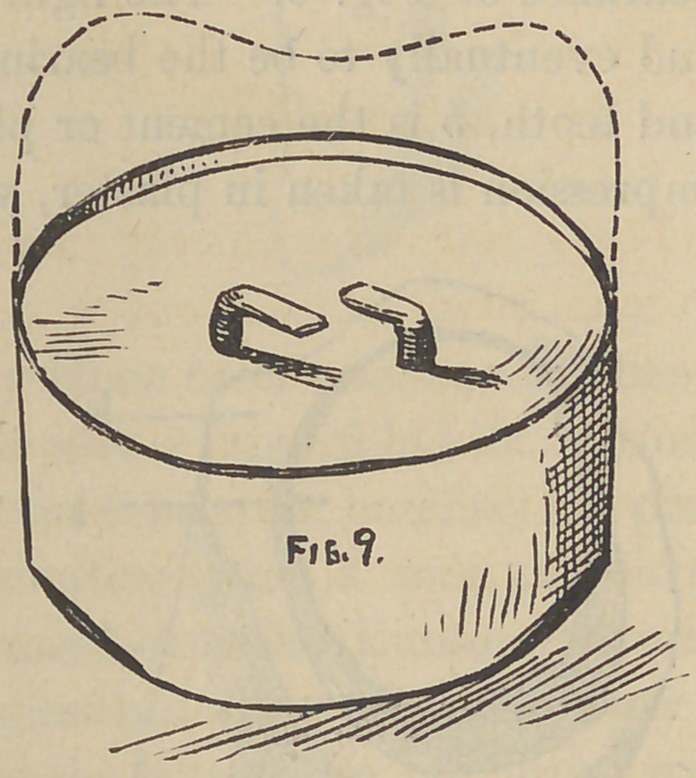


**Fig. 10. f10:**
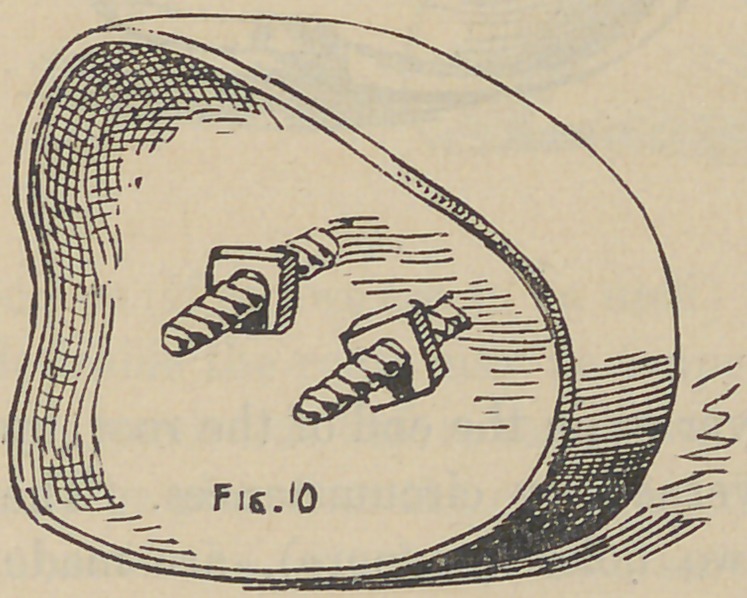


**Fig. 11. f11:**
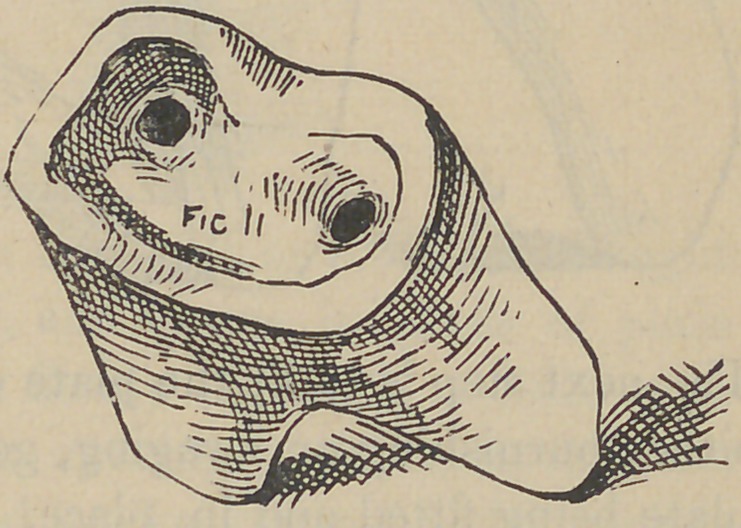


**Fig. 12. f12:**
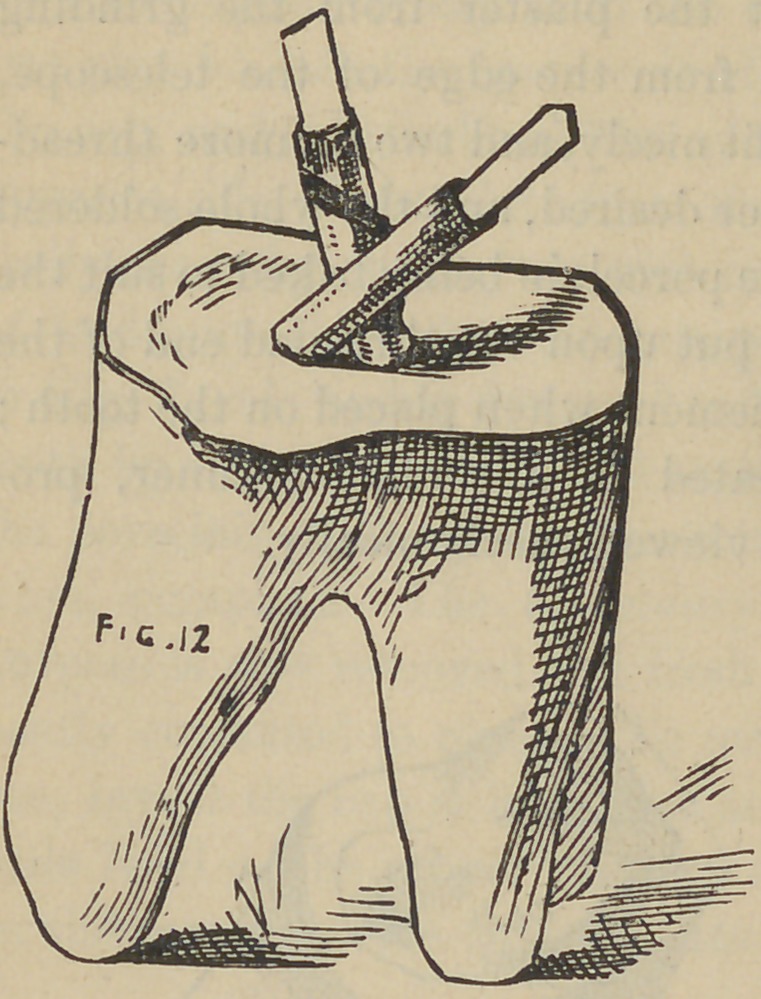


**Fig. 13. f13:**
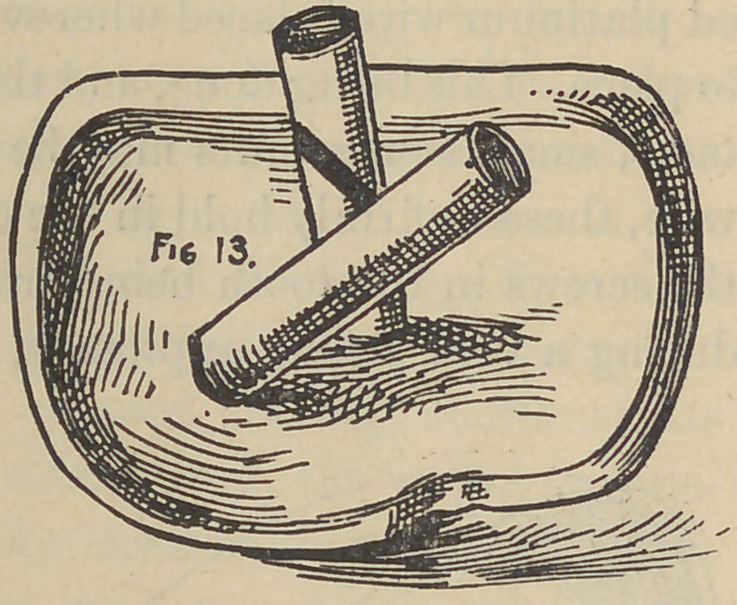


**Fig. 14. f14:**